# Health care providers’ perceptions of barriers to perinatal mental healthcare in South Africa

**DOI:** 10.1186/s12889-021-11954-8

**Published:** 2021-10-21

**Authors:** Shelley Brown, Courtenay Sprague

**Affiliations:** 1 Department of Health Sciences, Sargent College of Health and Rehabilitation Sciences, Boston University, Boston, MA USA; 2 Department of Conflict Resolution, Human Security and Global Governance, McCormack Graduate School of Policy and Global Studies, University of Massachusetts Boston, Boston, MA USA; 3 Center for Peace, Democracy and Development, Department of Conflict Resolution, Human Security and Global Governance, University of Massachusetts Boston, Boston, MA USA; 4 Department of Nursing, College of Nursing and Health Sciences, University of Massachusetts Boston, Boston, MA USA; 5 Faculty of Health Sciences, University of the Witwatersrand (Wits), Wits Reproductive Health and HIV Institute, Johannesburg, South Africa

**Keywords:** Perinatal mental health, Healthcare, Social determinants of health, Mental health services, Health systems, South Africa

## Abstract

**Background:**

Perinatal mental disorders are a leading contributor to morbidity and mortality during pregnancy and postpartum, and are highly treatable when identified early. However, many women, especially in low and middle-income countries, lack access to routine identification and treatment of mental illness in public health settings. The prevalence of perinatal depression and anxiety disorders, common mental disorders, is three times higher for South African women relative to women in high-income countries. The public health system has begun to integrate mental health into maternal care, making South Africa a relevant case study of perinatal mental healthcare. Yet studies are few. We sought to investigate healthcare providers’ perceptions of the barriers to early identification and screening of common perinatal mental disorders in public health facilities in South Africa.

**Methods:**

Employing qualitative methods, we used purposive sampling to identify study participants, supplemented by snowball sampling. From September 2019–June 2020, we conducted in-depth interviews with 24 key informants in South Africa. All interviews were recorded and transcribed verbatim. We used a thematic approach to generate initial analytical themes and then conducted iterative coding to refine them. We adapted a delivery systems’ framework to organise the findings, depicted in a conceptual map.

**Results:**

Reported barriers to early identification and treatment of mental illness in the perinatal period encompassed four levels: (1) structural factors related to policies, systems and resources; (2) socio-cultural factors, including language and cultural barriers; (3) organisational factors, such as lack of provider preparation and training and overburdened clinics; and (4) individual patient and healthcare provider factors.

**Conclusion:**

Barriers act across multiple levels to reduce quality mental health promotion and care, thereby creating an environment where inequitable access to identification of mental disorders and quality mental health services was embedded into systems and everyday practice. Integrated interventions across multiple levels are essential to improve the early identification and treatment of mental illness in perinatal women in South Africa. We provide recommendations derived from our findings to overcome barriers at each of the four identified levels.

**Supplementary Information:**

The online version contains supplementary material available at 10.1186/s12889-021-11954-8.

## Background

While the Sustainable Development Goals (SDGs) recognise the importance of mental health, perinatal mental ill-health continues to pose a global public health challenge, and COVID-19 has exacerbated matters [1–4]. Increasing attention has been paid in recent years to the significance of perinatal mental health throughout the life course, including negative health impacts of perinatal mental disorders on the foetus, infant, child and woman [2, 5]. The prevalence of common perinatal mental disorders (CPMDs) varies by country, but is generally higher in low and middle-income countries (LMICs)—15.6–19.8%—relative to high-income countries (HICs), where prevalence is 10.0–13.0% [6–9]. Perinatal mental disorders are a leading contributor to morbidity and mortality during pregnancy and postpartum and are often highly treatable when identified early. Yet, many women lack access to routine identification and treatment, especially in LMICs [2, 6].

South Africa is a highly relevant context to investigate perinatal mental healthcare as South African women have almost three times the prevalence of CPMDs, which includes major depression and anxiety disorders, compared to women in HICs. Research in South Africa indicates that 32.0–47.0% of antenatal women met screening criteria for depressive symptoms [10–12]. Additionally, the socio-economic and cultural context combine with the high burden of HIV, intimate partner violence, food insecurity, and an increasing non-communicable disease (NCD) burden, to exacerbate poor mental health in this population [13–17].

In order to improve maternal and child health, experts recommend integrated interventions to address multiple risk factors, including integration of mental healthcare into maternity services [10, 18–21]. A key component of the integration of mental health into maternity care in South Africa is the early identification of CPMDs in women, typically through the use of a brief symptom questionnaire, ideally combined with psychosocial risk screening and, when possible, an understanding of context-specific local idioms of distress [7, 18, 22, 23]. Yet the majority of antenatal and postpartum women rely on the under-resourced South African public health sector, utilised by 84% of the total population [24]. Further, public mental health spending has accounted for only 5% of the total public health budget [24, 25, 10]. As such, it has been a struggle to ensure women are routinely screened for CPMDs and practices vary widely by clinic [25]. Moreover, there is an unequal distribution of healthcare workers and shortage of staff that is felt most acutely in nursing services [26, 27]. Additionally, researchers have documented that some women experience disrespectful maternity care, especially marginalised women from lower socio-economic backgrounds [28].

Good governance of mental healthcare includes providing the necessary policy and legislative framework to promote and protect mental health of populations, while ensuring the optimal implementation of policies and practices that promote equitable health systems [29–31]. South Africa has demonstrated a strong government commitment to supporting the mental health of their population, in part through the National Mental Health Policy Framework and Strategic Plan (2013–2020) [32, 33]. The plan is well-intentioned and progressive, focusing on multisector collaboration and integration of mental health into primary healthcare through a task-shifting or sharing approach [34]. However, despite a promising policy framework, the governance of mental health in South Africa has been inadequate to date, in part due to a well-documented absence of meaningful implementation of mental health legislation and policies [35, 36]. The policy environment in South Africa mandates integrated perinatal mental health care, including screening, for perinatal women, but the lack of translation of national policy into sub-national, regional and local level policies reduces access to services and creates inequities in early detection, referral, education and treatment [22, 37]. Additionally, the insufficient preparation of non-specialist providers to deliver mental health screening and interventions has also hindered the timely delivery of quality mental healthcare [34, 38]. The Lancet Commission on global mental health and sustainable development views the SDGs as a strategic opportunity to improve mental health for whole populations, including a focus on social determinants and environmental influences on mental illness [39]. However, this requires greater evidence of health system responses to perinatal mental health in LMICs to inform research and policy [31]. This research seeks to contribute to filling this gap by documenting barriers to perinatal mental healthcare in the public health system reported by healthcare providers in South Africa. Our research question was: what are healthcare providers' perceptions of the barriers to early identification and screening of common perinatal mental disorders for women in public health facilities in South Africa?

## Design and methods

We employed a qualitative design and methods to explore the complex nature of barriers to mental health services. Qualitative methods allow for building trust with key informants and fostering a meaningful research relationship to better ensure the trustworthiness and reliability of qualitative data [40]. The research conformed with the Consolidated Criteria for Reporting Qualitative Research (COREQ) Checklist. We included a detailed account of the COREQ checklist, as applied to our research, in Table A of the Supplementary Materials section [41] .

### Sampling participants

We used purposive sampling to identify initial key informants for the study, supplemented by snowball sampling to locate additional key informants [42]. We selected eligible key informants based on three criteria: 1) knowledge about the concern under investigation; 2) willingness to discuss it; and 3) representation of a range of perspectives [42]. We recruited English speaking key informants via an email invitation that included detailed information about the study.

### Ethics

We received ethics approval from the Institutional Review Board of the University of Massachusetts Boston. All key informants provided verbal informed consent for study participation and digital recording. We maintained key informants’ confidentiality and anonymity throughout. We stored digital recordings and data in an encrypted, password-protected, secure location requiring authentication.

### Data collection

A total of 24 key informants with expertise in maternal health and/or mental health agreed to participate. Data were collected through in-depth interviews using a semi-structured question guide to capture key informants’ perceptions of barriers to perinatal mental ill-health identification and treatment in practice. The interviews were conducted by the first author, from September 2019–June 2020 via Zoom (20); telephone (2) and Qualtrics (2), an online survey tool. The same question guide was used for all data collection modes. The question guide included four main categories of questions related to: the perinatal mental health landscape and service delivery; mental health policies and practices concerning screening and early identification of CPMDs; implementation of mental health policies; and recommendations for addressing barriers to implementation. Key Informant interviews spanned 45–80 min in duration. We conducted interviews until saturation was reached, i.e., no new information emerged of significance to the study aim [42].

Research has indicated that some participants prefer the use of Zoom to in-person interviews, as the benefits of Zoom include establishing rapport, convenience and user-friendliness [43]. This accurately characterises the researchers’ experiences in the current study. Additionally, Qualtrics was found to be a useful data collection tool as an alternative to Zoom for two respondents. They worked in busy clinical settings during the early days of the COVID-19 pandemic and thus communicated their interest in participating and their preference for the flexibility of the online survey format, in lieu of a scheduled, longer Zoom interview. These realities highlight the preferences for remote research interviews during COVID-19 among respondents based in clinical settings. Nonetheless, preferences might differ for other types of participants, work settings and at other times.

The key informants included four medical doctors (three of them obstetricians); four psychologists; four mental health counselors/social workers; three psychiatrists (two focused on perinatal psychiatry); three birth and postpartum doulas; three nurse-midwives; and a government maternal health professional. We also interviewed two mental health academics, one with a clinical background in nursing, to gain their policy perspectives. All key informants were based in South Africa, working, or with extensive expertise in, maternal mental healthcare in the public health system.

### Data analyses

**Fig. 1:**
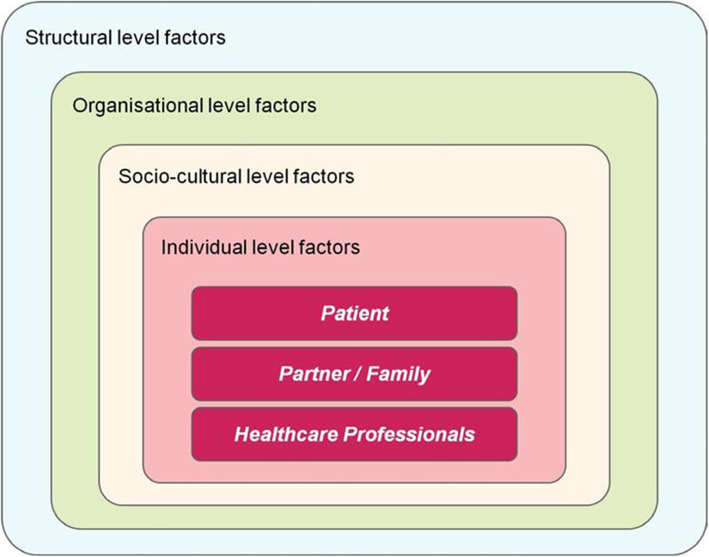
Adapted model depicting multilevel conceptual framework for barriers to mental health services in the perinatal period [44]

We listened to the recordings and transcribed them verbatim. We then reviewed the transcripts and began open coding, identifying emerging themes and related subthemes on healthcare provider perspectives. The authors met frequently to discuss the emerging themes, revise and refine them. After we identified and developed the themes, we then mapped the findings onto the theoretical multilevel conceptual framework, adapted from previous delivery systems models, (Fig. 1), originating from a systematic review that examined barriers to mental health service access for women with perinatal mental illness [44]. To ensure quality, rigour, and reliability in data collection, analyses and reporting, we maintained an audit trail, kept field notes, used purposive sampling, relied on thick description that retains the context of the data, employed a code-recode strategy, peer debriefings (discussion) and reflexivity among the co-authors [45].

**Fig. 2:**
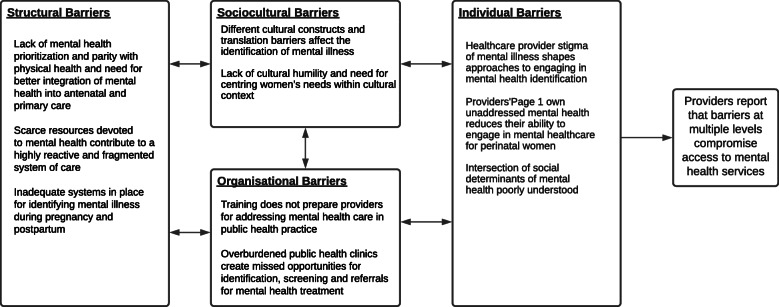
Conceptual map of barriers by level that impact early identification and screening for perinatal mental health services in South Africa

The conceptual framework organised and elucidated themes and subthemes on healthcare provider reported factors—occurring at the individual, organisational, sociocultural and structural-level—that were perceived barriers to early identification of perinatal mental illness cited by respondents. We then performed a cyclical, iterative review of themes and subthemes to further refine the “story” of the data (p. 87), represented in the adapted conceptual map (Fig. 2) [44, 46].

## Results

The study aimed to understand healthcare providers’ perceptions of barriers to mental health services, including early identification of mental ill-health, and the reported challenges their patients faced. Based on the multilevel model [44], we organised the findings into the following levels: Structural (unclear policy); Sociocultural (family support, social support networks and cultural attitudes); Organisational (organisational characteristics, service access and inadequacy of resources) and Individual (knowledge, attitudes and individual characteristics of women, their families and healthcare providers) [36, 44]. Our results indicate that barriers to early identification of mental illness (and accessing mental health services more broadly) exist at many levels. In the next section, we present the findings, employing illustrative quotes by level and subtheme. To reflect the key informant narratives and tell the story of the data, we begin with organisational-level factors.

### Organisational

#### Training does not prepare providers for addressing mental healthcare in public health practice

Respondents described a mismatch between didactic, in-service training contrasted with the ‘grounded’ realities of clinical public health practice. A psychiatrist highlighted the lack of prioritisation of mental health in medical school rotations. Psychiatric rotations in South Africa often occur in the final year of medical school and are typically briefer than other rotations. She, like others, considered this preparation insufficient since most doctors will encounter patients experiencing mental disorders frequently in practice:

*[I]n your casualty or emergency department [rotation], you'd find that 50% of patients have some sort of psychiatric issue, emergency psychiatric issue…the training doesn't speak to what is happening on the ground. (Key informant 21)*

In addition to the lack of emphasis on psychiatry in medical school, there is currently no specialisation in perinatal psychiatry. Psychiatrists identified this as a gap in their preparation and training, noting,

“*there's no specific qualification [perinatal mental health qualification for psychiatrists] here yet, so everyone just does their own thing, and then we meet up and say we all do it. There are a couple of us in the country who are interested in it, but it's not as organised as it is in the private sector. (Key informant 20)*

Equally, a Director of Nursing Education highlighted a gap in the nursing curriculum and preparation. She emphasised the need for critical thinking approaches that also incorporate social determinants of health into clinical practice:

*How can you have empowered students that are critical thinkers when your lectures aren’t critical thinking? How do you get that critical engagement with those issues [underlying social determinants of health] and realisation that you can't just send people back to where they came from, and give them a nice little disconnected lecture on various things that they're going to do, and then send them back to where all the problems are? It is a challenge to translate that in a meaningful way in a curriculum, especially when they also work in the practical environments where people come from very traditional training... (Key informant 9)*

The Director just cited underscored a lack of training to address the ‘real world’ needs of women, including social determinants of health that affect them within their family and social contexts.

Key informants reported that the dearth of preparation in medical and nursing school and lack of specialization sets a poor foundation for healthcare providers entering clinical practice: i.e., once in practice, training to identify, refer and treat women with CPMDs remains inadequate. A medical doctor confirmed the need for more effective training, but emphasised challenges in the feasibility of implementing and sustaining such training:

*I think there is a massive need for training providers to engage in mental health during ANC visits...my hesitancy comes from how you practically do that? Taking people out for training means that there's less people to provide services, which is a constant challenge that we find with anything that we try to implement. It takes training and also follow up mentorship and support. (Key informant 22)*

#### Overburdened public health clinics create missed opportunities for identification, screening and referrals for mental health treatment

Most key informants commented on the busy clinic environment not being conducive to engaging in discussions about mental ill-health. As one key informant commented, nurses have so many patients, they will rush through an hour-long visit in 15–20 min—neglecting the opportunity to screen for CPMDs or discuss current experience of distress with women, thus posing missed opportunities for addressing mental ill-health during the visit. Further, the key informant suggested that a woman in distress that is experiencing mental ill-health might not return, even if a subsequent visit is scheduled for her.

*…if it's one nurse and she has 30 or 50 new first visit ladies that's announcing their pregnancy for the first time and because of our HIV burden the first visit should take you an hour. But that's not the only burden, there’s another 60 [patients] coming for their second and third visits. And she will try and rush through this first visit in about 15 or 20 minutes. And the lady will get a second visit date, but she's mentally not well so she will never pitch up again, you see, and then that's a missed opportunity and that’s exactly what we have. (Key informant 10)*

Psychiatrists and other mental health specialists typically only see women when they are in late stages of pregnancy and have more severe mental illness, with little time to intervene prior to childbirth. A psychiatrist noted that this exposes the gaps in the system:

*…a lot of these women, you can see where the gaps are coming in. By the time they reach us, a lot of them get to us at late gestations and we don't have much time to sort them out and you can see where they [have] fallen through the gaps along the way because of the overburdened clinics that they're coming from. (Key informant 22)*

One key informant suggested that a simple question, “How are you?” might begin to engage women in conversations about their mental health. Yet clinics are usually too burdened to allow for much time with the patient, so providers often avoid the conversation entirely.

*Some of these women they feel quite isolated so just asking them how they are and just the brief discussion would alleviate [mental health issues], especially in the milder forms. …but for the midwives, it’s so pressured. They will see 35 women in the waiting room…not having the time to spend with the women. I think they avoid that [discussion of mental health]. (Key informant 5)*

Long queues and overwhelmed staff in many antenatal clinics further impede women from asking questions of providers during visits. Due to long queues, one provider shared an example where a woman struggling with depression would not be comfortable disclosing concerns about her mental health, even if she desired it.

*I'm not going to go to the doctor and start talking about my emotions and how I'm feeling, because we've been waiting since five in the morning. And there are 100 other people…outside. So they're not going to easily say “Hey Doc, you know, things aren't going well.” …and then the doctors aren’t asking the questions on their end, everything just kind of gets messed up. (Key informant 21)*

### Structural

#### Lack of mental health prioritization and parity compared with physical health and need for better integration of mental health into antenatal and primary care

Respondents discussed the lack of streamlined care and integration of mental health into ANC as part of the challenge of meaningfully addressing women’s mental health.

*It’s very difficult for these women to have a separate health visit for mental health…because you've got to take time off work. You’ve got to organise childcare. You've got to pay for traffic transport, get to the clinic, see the doctor and then do all your antenatal visits…it really needs to be done all at the same time. It takes a lot of motivation to take the two taxis with your toddler to sit in on the line at another clinic to see someone that may or may not help you. (Key informant 23)*

Deploying community health workers to address mental health, as part of a task-sharing approach, is one health system innovation to reach more women and better integrate mental healthcare. And yet a psychiatrist stressed that the overwhelming and unrealistic caseloads faced by community health workers lessens their ability to care for women’s mental health.

*… you read their job description and you just think how on earth are they supposed to do that … for 250 households…it's insane. Maybe a community healthcare worker could manage 50 households, if 40 of them are stable. It’s all very nice, in theory, on paper, but when you try and practically implement it, you realise that these poor community healthcare workers. There's no way that they can handle all of this. (Key informant 22)*

#### Scarce resources devoted to mental health contribute to highly reactive and fragmented system of care

Competition for public health resources occurs at the highest levels and this reduces the attention that mental health receives, as one key informant related.

*At the macro level, I think in South Africa we have a situation of enormous competing for the public health crises…in terms of not only the obvious things like funding and programmatic attention, but they compete psychologically in people's mind. [There are] challenges which are enormous, like gender-based violence and corruption and food insecurity and a lack of access to basic resources, and it just feels that mental health is not as tangible. The average high-level minister or politician or person who allocates budgets… I think it just must be very difficult for them to keep all of these things in mind and to have a concurrence. (Key informant 3)*

As noted by many key informants, South Africa’s mental health service delivery system, like many other settings, is highly reactive, not anticipatory. One key informant described this culture in the following way.

*…part of the issue in South Africa is the translation of all these wonderful ideas into reality is where it falls down. And what you find…on the ground is very different to what we might be on paper, and I just think that the services and the service delivery that we have in South Africa is highly reactive and there is no probing to and educational work being done. Mental health services are reactive and when there's a problem. (Key informant 19)*

Key informants expressed frustration, indicating that it is not enough to provide mental health education for women in the community when the health system is weak. Some key informants called for a better system for all types of healthcare providers to work together to improve women’s mental health.

*It can't just be one person, it needs midwives, it needs psychologists, it needs lactation specialists. It needs the paediatricians, obs and gynae. All of us need to work together…I decided it's no use me going on all these platforms and educating the community and then when they get to the antenatal clinic there's absolutely no services available or no one knows what they're* [the patients] *talking about. (Key informant 21)*

#### Inadequate systems in place for identifying mental illness during pregnancy and postpartum

There are emerging systems, including a brief, validated screening tool, for early identification of common perinatal mental disorders, as one provider noted, “*It is however in the primary healthcare guidelines…it’s not routinely done yet, but it should be because the primary healthcare guidelines advocate for mental health to be incorporated into the antenatal clinic. (Key informant 23).”* However, rather than being delivered routinely, many key informants portrayed an environment where early identification of mental disorders depended on the ability or willingness of providers.

*How much it practically filters down into the clinics [mental health screening], I think it depends on the sort of clinician who maybe has an interest who sort of pick up the cues on a patient who has got issues. The size of our clinics and probably just the interest of people. I don't think it's screened for enough. (Key informant 18)*

A psychiatrist noted a failure of early screening, which leads to seeing patients with more complex issues later:

*Specifically, a lot of the postpartum stuff we don't pick up…it only presents when you take a history a couple of years later, like what happened then… that initial screening, that postpartum screening. I don't think it's being done accurately by gynaes, you know they're supposed to do that, at the 10 day visit, six week visit. I don't think it's being picked up, but I also don't think women are aware. And these are kind of first onset stuff. It also kind of depends on which gynae you have, I think. If a gynae is more psychologically minded, they will ask more. (Key informant 20)*

### Sociocultural

#### Different cultural values and translation barriers affect the identification of mental illness

One key informant mentioned that nurses will record in a patient’s file that a patient is “uncooperative” if they don’t speak the same language or understand directions they are given in the clinical setting. She indicates this notation in the file follows the patient, who might be treated poorly throughout her time in care.

*Instead of somebody sitting down and thinking maybe she's just scared or maybe she doesn't actually understand what's going on because the language barrier is just as bad… First of all, she's really intimidated by what's going on, and in the black cultures going in and having somebody A) that’s very educated and B) that’s a senior member of your community then coming in and treating you so badly, it just breaks down your entire self-esteem. So you think to yourself….I am a terrible, terrible person. (Key informant 12)*

One respondent described challenges directly translating depression and anxiety, also observing that some patients’ experiences of distress manifested as physical symptoms.

*If you say you're depressed, there isn't a direct translation for example for depression or anxiety …patients will often somatise and say… in Xhosa “I have a headache or it’s my back”…and you’ll treat that headache to death. Because it’s culturally more appropriate to go to the doctor or nurse and say, I have a headache* [than to admit to depression]*. (Key informant 21)*

A different key informant discussed how local understandings of depression and idioms of distress were not readily translated to conform with commonly understood definitions of depression. The respondent suggested that the lack of understanding of local idioms of distress, combined with low community level awareness of symptoms of mental ill-health, limited healthcare provider identification of CPMDs, like depression. Ultimately, these expressions of distress might be ‘normalised’ as simply part of the experience of a difficult life and not understood as mental ill-health.

*If I try and ask women directly about depression, very few of them even know what that means. So then I ask a nursing sister to come and translate it into the vernacular language so that they can have a sense. Listening to those translations, I get the sense that there isn't even a word for depression in many of the South African vernacular languages so I think it's not something that's widely acknowledged in the community. And I think most women would probably think it's pretty normal to be miserable about life because life is tough. I'm not sure that women would realise that low mood or suicidal thinking or finding it hard to bond with your baby or not feeling excited about the pregnancy is unusual or abnormal. (Key informant 22)*

#### Lack of cultural humility and need for centring women’s health within the cultural context

Respondents continued to stress that women might use less specific terms or clues that indicate they are not doing well and are sad, and the respondents reinforced that clinicians need to shift to focus on the holistic health of women, to understand different manifestations or expressions of mental ill-health in perinatal women, while keeping women at the centre.

*… some people will come in and they'll talk about poor sleep and poor appetite and poor memory and concentration and anhedonia but they don't come in and say I'm feeling actually really sad. (Key informant 23)*

A doula who supports women during childbirth observed that women are routinely discouraged from asking providers questions about their health. She suggested that if providers were reminded of the importance of putting patients’ needs first, in accordance with *Batho Pele* principles (‘People First’ in Sesotho), rather than solely focusing on routine requirements in a ‘robotic’ manner, this could be an important step in opening the dialogue about mental health [47]. The intent of *Batho Pele* includes transformation service delivery in the public sector; increase access and generally ensuring good customer service.

*Providers need to be reminded about the importance of individual patients. It’s crazy because it’s your body. We feel like it’s so hard to ask that question. You are not questioning their knowledge, in fact you want them to share more with you. Often you will find when you ask the provider, they are humans too. Sometimes they have to do something in a robot way…they need a little reminder that they need to take a moment with someone. (Key informant 7)*

### Individual

#### Healthcare provider stigma of mental illness shapes approaches to engaging in identification

Key informants observed the mismatch between the content of the current training and that required to address the mental health needs of perinatal women, mentioning that the insufficient attention to mental health in training serves to stigmatise and undermine the importance of mental health:

*That in itself [insufficient training on mental health] already cements mindsets and stigma with regards to the importance of mental health. (Key informant 21)*

Key informant 21 elaborated her view of the cultural construct and stigma of mental illness and the implications of poor mental health in communities, suggesting that this affects how mental illness is viewed by individuals and providers alike:

*South Africa is 80% black population and culturally, if we look at it from…an African point of view, there is no such thing as mental illness. So we already have that stigma. So it’s stigma within medicine itself and the perceptions of psychiatry, and then you have a cultural stigma as well. (Key informant 21)*

A doula shared her observations of stigma at the individual-level in South Africa, as shaping perinatal women’s interactions with their providers, *“It’s hard because there’s such a stigma especially when you’re a new mother, it’s so hard to say “I do need the help.” It’s a great tragedy of our time that women have the burden. The mental load of everything the mother needs to think. (Key informant 7).*

Similarly, a perinatal psychiatrist emphasised that stigma is present at multiple levels.

*…we've got stigma at all different levels. We’ve got stigma at…the midwives, who have called the patients crazy literally. We've got [self] stigma at the patients themselves, not wanting to say that they're struggling. And then you know, there's this whole “go to psych, go see psych” and it's a very derogatory statement and even like in the clinics where I used to work…the psych section was the bad section. (Key informant 20)*

Key informant 20 indicates that stigma acts in multiple ways to compromise providers’ ability to detect and treat mental ill-health during ANC visits.

#### Providers own unaddressed mental health reduces their ability to engage in mental healthcare for perinatal women

A doctor with extensive clinical and research experience in maternal health depicted a context where nurses typically come from the same communities as their patients, and thus face similar challenges.

*If you look at the sort of predictors of the patient and …social circumstances of the pregnant women…nurses have the same predictors. And then you match the two, and you have a woman that's down and you have a nurse that's down and there’s no care happening. They miss the biggest things that you even can think about. (Key informant 10)*

Key informant 10, and others, indicated that providers’ unaddressed mental health needs appeared to limit their capacity to engage women in identification, referral and treatment of CPMDs. Indeed, many key informants emphasised that the frequency of unaddressed mental health issues among health professionals rebounds to undercut their ability to engage their patients in mental healthcare. A psychiatrist explained how providers with experiences of unresolved trauma and mental illness are less likely to engage in mental health conversations with women because these might be a triggering experience.

*I think a lot of it comes down to your own personal experience and…past exposure to people who've had mental health issues. I think one of the big reasons it's not engaged in is because our staff have a high rate of mental health issues that are not addressed. We are always encouraging them, particularly in the antenatal clinics, to screen for gender-based violence. But if you're a victim of that yourself, then the triggers involved in screening other people for it are just so big…I'm just very aware of how much vicarious trauma and sort of personal trauma exists within the healthcare workforce that can make it quite difficult for them to then open up a can of worms in someone else. (Key informant 22)*

Similarly, another respondent noted that the absence of psychosocial support (referral services) reduced the emotional capacity of providers. This, together with the high burden of patients’ overall needs, signified that providers might be less likely to address the mental health needs of women.

*And then what do you do when you're sitting with an awareness that a patient has a problem, but you've got nowhere to divert them to and you don't have the skills or the time or the emotional capacity to deal with it. It's much better to just not go there. Safer for you to not go there. And I think there's quite a lot of sort of hardness amongst some healthcare workers because that's their survival option…just shut off and don't connect with the humanity in the person that you're seeing, because if you do, you're just never going to make it through the day, there's so many women with so many challenges that you would…only get through five people in a day, and you've got 73. (Key informant 22)*

This ‘hardness’ seemed to serve as a sort of coping strategy for providers, given the absence of resources, training and emotional support for themselves.

#### Intersection of social determinants of mental health poorly understood

A doctor with many years of experience in perinatal mental health stated that the multiple, intersecting social determinants of mental health are poorly understood, jeopardise overall well-being of patients, and seem to reduce expectations of what providers offer women.

*We are finding that they're not facing one or two risk factors, they are facing five or six, in their histories or in their current life circumstances. So we've had to kind of adjust expectations and not necessarily expect that women will end up being completely well and completely flourishing. It won't solve the fact that they've got no running water, or…that their education means that they will only earn 3000 rand a month [approximately 163 Euros] if they're lucky. So we've had to get comfortable with our limitations and try to work within that. (Key informant 3)*

In response, one doctor discussed working within these limitations to provide a brief mental health screen, which she observed could make a meaningful difference in the face of these intersecting risk factors and determinants of mental health.

*It's almost like people perceive there to be bigger problems [than mental health]. But I don't think people understand that a woman who is depressed is more likely to be in an abusive relationship, is more likely to be exposed to HIV. There's that triad. So all those are all interlinked. …you sort out her mental health, you might actually put her at less risk of HIV, or you might make them healthier so that she can actually comply with her ARVs and be healthy. But I think,…we're so used to putting out fires. (Key informant 23)*

Several key informants mentioned that these complex, interlinked factors contributed to an assumption among providers that depression and anxiety are, again, ‘normal’ responses to life’s challenges in South Africa. They explained that this normalisation contributes to women suffering in silence.

*…you're looking at almost 40% of women having postnatal or antenatal depression, anxiety, it’s not recognised because it’s so common. …one of the gynaes [gynaecologists] was saying to me: “Well, obviously, everyone's depressed. No one's got partners, no one's got money, you know, everybody's HIV positive...no wonder they’re feeling depressed.” (Key informant 23)*

Respondents highlighted, per previous comments, that doctors, nurses and midwives often race through checklists and miss important disclosures, such as intimate partner violence or food insecurity, emphasising that centring the conversation around the holistic needs of the individual women is needed. As one provider elucidated,

*…you must ask a patient what matters to them. They have a certain reason why they want to come to you. And that is one of the things that you need to address because the patients will actually tell you when they're not well, but if you don't give them the opportunity, you will never hear it. A pregnant woman will tell you that she's very worried about the baby, that it’s not going well, but actually, the husband is being violent or there's not enough food at home, and now the baby's not growing well…and if you go through a checklist, like a robot, you will not hear this. (Key informant 10)*

Key informants were asked during the interviews if they had any specific suggestions concerning how perinatal mental health policies could be improved, and they eagerly shared potential interventions and recommendations to overcome barriers by level (Table 1).

**Table 1:** Recommendations to address barriers to early identification of CPMDs

Level	Recommendations
Structural	• Provide guidelines for health professionals providing maternity care services on best practices for routine screening of CPMDs in maternity care settings. • Expand MOMConnect mobile app mental health messaging supported by the National Department of Health [48]. • Continue expansion of psychosocial support via NurseConnect mobile health application supported by National Department of Health [49]. • Identify lessons learned from the CLEVER Maternity Care intervention, which aims to increase respectful, quality obstetric care [50].
Sociocultural	• Increase awareness and availability of support from doulas: trained professionals who provide culturally competent, continuous physical, emotional and informational support to mothers before, during and shortly after childbirth. • Train perinatal care professionals in cross-cultural care and humility.
Organisational	• Designate clinic days devoted to mental health where dedicated perinatal mental health counselors are available to reduce stigma and avoid wait times typical of referral processes. • Add required continuing education centred on social determinants of mental health and intersecting risk factors. • Review how and when mental health is promoted and offered in health professions curriculum. • Address issues underlying the chronic nursing shortage in South Africa and review approaches to task-sharing nurses’ roles with community health workers
Individual	• Improve mental health training to recognise underlying determinants of health that shape physical and mental health outcomes, especially for antenatal and postpartum women. • Increase Employee Wellness opportunities for health professionals to seek counseling, support and opportunities to debrief. • Expand peer mentorship programs, like The Mentor Mother programme, where peers are available to empower other women during pregnancy and breastfeeding, particularly for women without partners [51].

## Discussion

The study explored healthcare providers’ perceptions of barriers to early identification and screening of CPMDs. The findings captured complex multilevel factors that shape women’s access to mental health services, as reported by healthcare providers. Drawing on previous research and the multilevel conceptual framework, we developed a conceptual map (Fig. 2) to depict the barriers reported by healthcare providers in South Africa and how they influence early identification of mental illness and mental health services [44].

Providers shared that **organisational factors** affected the quality of care received by perinatal women, through lack of training, preparation and overburdened clinics. Key informants reported that insufficient training in understanding the significance of mental health in clinical practice paired with overburdened public health clinics, created missed opportunities for identification, screening and referrals for mental health treatment. Key informants reinforced that a mental health screen during ANC visits could make a difference in the trajectory of a woman’s well-being, especially if she is experiencing distress, and that follow-up with a specialist is needed. Research suggests that perinatal women and healthcare providers believe that screening for CPMDs is acceptable, and that this is important for effective implementation of screening policies [22, 48]. Due to insufficient numbers of trained mental health professionals, South Africa has focused on task shifting to non-specialist provider delivery of perinatal mental health screening [22]. Despite the acceptability of routine screening, and the task-shifting approach embraced within South Africa, without adequate training, supervision and mechanisms to prevent overburdening staff, missed opportunities for identification and referral for mental health treatment will persist.

**Structural factors**, including lack of mental health prioritisation, parity with physical health, and the need for better integration of mental health into antenatal and primary care emerged as a dominant theme. These factors created an unstable foundation for meaningful integration of perinatal mental health into primary care. Researchers in one study of Xhosa-speaking pregnant women in Cape Town similarly found that many symptoms experienced by women are related to “external life stressors,” many of which are out of the control of women and rooted in a history of discriminatory policies [23]. That study emphasised the importance of incorporating research on mental health into policy development to begin to address these structural risk factors [23]. Researchers have also highlighted the limited public health system expenditure on mental health services, which ranges by province, and the role this plays in producing inequities and inefficiencies [26]. Inequities within and among facilities and provinces include lack of access to psychiatrists and mental health specialists. Due to the dearth of mental health specialists, nurses have been recognised as the “backbone” of primary healthcare service integration of mental health service delivery [26]. Evidence points to the need for increased resources for nurses, community healthcare workers and other non-specialist providers to support the integration of mental health service delivery within the public health system [26].

**Sociocultural factors** reported by providers centred on the effect of different cultural constructs of mental illness for patient and providers, coupled with language and translation barriers, thus limiting the identification of CPMDs in perinatal women. Some key informants noted that a lack of cross-cultural understanding contributed to missing key signals when patients described physical manifestations (like a headache) of depression or anxiety that did not align with a provider’s perception of CPMD symptoms, and these respondents reported witnessing women treated poorly due to resultant misunderstandings. Similarly, research has demonstrated the importance of understanding local idioms of distress, symptoms and perceived causes of perinatal depression to develop context-specific tools for intervention and assessment of maternal depression [23].

A few respondents pointed to a lack of cultural humility exhibited by some providers when engaging with patients, as well as a need for centring women and their holistic health requirements during visits within the cultural context. In the absence of deeper cross-cultural understanding of women’s unique mental health needs, respondents believed that women might interpret their low mood as a ‘normal’ part of the perinatal experience.

**Individual-level factors** also emerged as a salient theme, encompassing provider-driven issues of mental illness stigma and providers’ own unaddressed mental health. Healthcare providers reported a compromised ability to engage in identification of mental ill-health in patients due to the stigma of mental illness and their own unaddressed mental health issues, which reportedly constrained their capacity to screen women - even when guidance indicated they should routinely screen women for CPMDs. Unaddressed mental ill-health in providers shapes their ability and willingness to address mental ill-health in patients [52, 53]. Importantly, deeply embedded beliefs of providers towards mental illness affect how and why providers choose to screen for CPMDs and/or have conversations about mental health with perinatal women during patient visits [38]. Poor understanding of interlinked social determinants of mental health also operate to normalise depression and anxiety for perinatal women.

### Study limitations and future research

Limitations included a possible over-sampling and representation of key informant views from the better-resourced and urban provinces: Western Cape and Gauteng. The use of snowball sampling as a methodological approach relies on study key informants’ referrals to identify other key informants, and the small size of the perinatal mental health community in South Africa, coupled with this sampling approach, might have limited the type of key informants. A second limitation concerns the representativeness of the key informant population in the current study and the researchers’ selection of English language speaking key informants. There are 11 official languages in South Africa and English is not the most common language. Hence, future studies on perinatal mental health should consider the use of other languages in South Africa to better capture nuances in language and social meanings ascribed to specific words and phrases, including localised idioms and experiences of distress [23]. Additionally, two key informants completed a questionnaire via Qualtrics. While a uniform question guide was used throughout, the absence of researcher-led facilitation and interaction with the key informants in Qualtrics might have reduced thick description in two cases. The researchers acknowledge as an added limitation, the absence of triangulation of data sources, including qualitative interviews with perinatal women patients. Although this was originally intended, it was not possible. An important next step for research on barriers to early identification of CPMDs in South Africa is to include perceptions of perinatal women. Equally, future research on perinatal mental illness should aim to capture respondent views across provinces, including provider views from more rural and poorly resourced locations where identification and treatment barriers are likely to be highest.

## Conclusion

The absence of streamlined integration of mental health into primary care compounds the challenges of meaningfully addressing perinatal mental health to advance women’s health and wellbeing. Policies in South Africa have emphasised the importance of integrating mental health into antenatal and primary care. While well-intentioned, the approach to date has been ad hoc, fragmented and shaped by factors at the organisational, sociocultural, individual and structural levels. This fragmentation has created a series of missed opportunities. Findings here have illuminated factors across multiple levels that influence early identification of mental illness in perinatal women in South African public health facilities, and, ultimately, compromise women’s quality mental health promotion and care. Social determinants, including structural inequities, contribute to an increased risk for mental illness and shape access to mental health services. Hence, integrated interventions are needed to address multiple risk factors and at multiple levels to improve provider engagement in early identification and treatment of mental ill-health for perinatal women in South Africa.

## Supplementary Information

**Additional file 1: Table A**: Consolidated Criteria for Reporting Qualitative Research.

## Data Availability

The data produced and analyzed for the current study are not publicly available due to containing information that could compromise key informant consent. Data are available from the corresponding author upon reasonable request, however.
