# Supplementary material for: Health care providers’ perceptions of barriers to perinatal mental healthcare in South Africa
Source: BMC Public Health. 2021 Oct 21;21:1905. doi: 10.1186/s12889-021-11954-8 (PMC8528557; doi:10.1186/s12889-021-11954-8)
Supplement: Supplementary file 1 — Additional file 1: Table A: Consolidated Criteria for Reporting Qualitative Research. [file 12889_2021_11954_MOESM1_ESM.docx]

**Table A: Consolidated Criteria for Reporting Qualitative Research**

Adapted from: Tong A, Sainsbury P, Craig J. Consolidated criteria for reporting qualitative research (COREQ): a 32-item checklist for interviews and focus groups. Int J Qual Health Care. 2007;19:349–57.

| **Domain 1: Research team and reflexivity** | | |
| --- | --- | --- |
| 1. Interviewer | Which author/s conducted the  interviews? | SB |
| 2. Credentials | What were the researchers’ credentials? | SB (MPH) CS (PhD, joint-MA) |
| 3. Occupation | Researchers’ occupations at the time of the study? | SB, Clinical assistant professor of public health  CS, Associate professor of global health |
| 4. Gender | Were the researchers male or female? | Females |
| 5. Experience and training | What experience or training did the researchers have? | SB (8+ years of qualitative training and experience)  CS (Over 20 years of qualitative research experience) |
| 6. Relationship with participants established | Was a relationship established prior to study commencement? | All but one participant was unknown to the researchers. |
| 7. Participant knowledge of the interviewer | What did the participants know about the interviewer? | Participants knew the research rationale, study purpose and interviewer characteristics |
| 8. Interviewer characteristics | What characteristics were reported about the interviewer? Bias, assumptions, reasons and  interests in the research topic | The reasons for pursuing the research and interest in perinatal mental health shared with participants at start of interview |
| **Domain 2: Study Design** | | |
| 9. Methodological orientation and Theory | What methodological orientation was stated to underpin the study? | Qualitative paradigm and design |
| 10. Sampling | How were participants selected? | Purposive and snowball sampling |
| 11. Methodological approach | How were participants approached? | Participants were invited via email |
| 12. Sample size | How many participants were in the study? | 24 |
| 13. Non-participation | How many people refused to participate or dropped out? | No one refused to participate in the study |
| 14. Setting of data collection | Where was the data collected? | The data were collected in Boston, MA via Zoom, phone and Qualtrics with participants in South Africa |
| 15. Presence of nonparticipants | Was anyone else present besides the participants and researchers? | No |
| 16. Description of sample | What are the important characteristics of the sample? | Participants were all women; health care providers; researchers or key experts in perinatal health |
| 17. Interview guide | Were questions, prompts, guides provided by the authors? | Question guide described in methods section |
| 18. Repeat interviews | Were repeat interviews carried out? | No |
| 19. Audio/visual recording | Did the research use audio or visual recording to collect the data? | Yes; the researchers used audio and visual recording via Zoom |
| 20. Field notes | Were the field notes made during and/or after the interview? | After the interview |
| 21. Duration | What was the duration of the interviews? | 45-80 minutes |
| 22. Data saturation | Was data saturation discussed? | Discussed in methods section |
| 23. Transcripts returned | Were transcripts returned to participants for comment and/or correction? | No |
| **Domain 3: analysis and findings** | | |
| 24. Number of data coders | How many data coders coded the data? | 1 |
| 25. Description of coding tree | Did the authors provide a description of the coding tree? | No. Available upon request |
| 26. Derivation of themes | Were themes identified in advance or derived from the data? | Themes were derived analytically from the data after collection |
| 27. Software | What software, if applicable, was used to manage the data? | No data analysis software used |
| 28. Participant checking | Did participants provide feedback on the findings? | No |
| 29. Quotations presented | Were participant quotations presented to illustrate the themes/findings? Was each quotation identified? | Yes: quotations presented and identified |
| 30. Data and findings consistent | Was there consistency between the data presented and the findings? | Yes |
| 31. Clarity of major themes | Were major themes clearly presented in the findings? | Major themes were presented and described clearly in the findings |
| 32. Clarity of minor themes | Is there a description of diverse cases or discussion of minor themes? | Only major themes included; no diverse cases were applicable |
